# Supplementary material for: Styrene Production in Genetically Engineered Escherichia coli in a Two-Phase Culture
Source: BioTech (Basel). 2024 Jan 14;13(1):2. doi: 10.3390/biotech13010002 (PMC10801462; doi:10.3390/biotech13010002)
Supplement: Supplementary file 1 [file biotech-13-00002-s001.zip › biotech-2742900-supplementary.pdf]

Supplementary information

Sequence of AtPAL2:

ATGGATCAGATTGAAGCAATGCTGTGTGGTGGTGGTGAAAAAACCAAAGTTGCAGTT

ACCACCAAAACACTGGCAGATCCGCTGAATTGGGGTTTAGCAGCAGATCAGATGAAA

GGTAGCCATCTGGATGAAGTGAAAAAAATGGTGGAAGAATATCGTCGTCCGGTGGTT

AATTTAGGTGGCGAAACCCTGACCATTGGCCAGGTTGCAGCAATTAGCACCGTTGGT

GGTAGCGTTAAAGTTGAACTGGCAGAAACCAGCCGTGCCGGTGTTAAAGCAAGCAG

CGATTGGGTTATGGAAAGCATGAATAAAGGCACCGATAGTTATGGTGTTACCACCGGT

TTTGGTGCAACCAGCCATCGTCGTACCAAAAATGGCACCGCACTGCAGACCGAACTG

ATTCGTTTTCTGAATGCAGGTATTTTCGGCAACACCAAAGAAACCTGTCATACCCTGC

CGCAGAGCGCAACCCGTGCAGCCATGCTGGTTCGTGTTAATACACTGCTGCAGGGTT

ATAGCGGTATTCGTTTTGAAATTCTGGAAGCAATTACCAGCCTGCTGAATCATAACATT

AGCCCGAGCCTGCCGCTGCGTGGCACCATTACCGCAAGCGGTGATCTGGTTCCGCTG

AGCTATATTGCAGGTCTGCTGACCGGTCGTCCGAATAGCAAAGCAACCGGTCCGGAT

GGTGAAAGCCTGACCGCAAAAGAAGCATTTGAAAAAGCAGGTATTAGCACGGGCTT

TTTTGATCTGCAGCCGAAAGAAGGTCTGGCACTGGTTAATGGTACAGCAGTTGGTAG

CGGTATGGCAAGCATGGTTCTGTTTGAAGCAAATGTTCAGGCAGTTCTGGCAGAAGT

TCTGAGCGCAATTTTTGCCGAAGTTATGAGCGGTAAACCGGAATTTACCGATCATCTG

ACCCATCGTCTGAAACATCATCCGGGTCAGATCGAAGCAGCAGCCATTATGGAACATA

TTCTGGATGGTAGCAGCTATATGAAACTGGCACAGAAAGTTCATGAAATGGATCCGCT

GCAGAAACCGAAACAGGATCGTTATGCACTGCGTACCAGTCCGCAGTGGCTGGGTCC

GCAGATTGAGGTTATTCGTCAGGCAACCAAAAGCATTGAACGTGAAATTAACAGCGT

GAATGATAACCCGCTGATTGATGTTAGCCGTAATAAAGCAATTCACGGTGGTAATTTTC

AGGGCACCCCGATTGGTGTTAGCATGGATAATACCCGTCTGGCAATTGCAGCCATTGG

TAAACTGATGTTTGCACAGTTTAGCGAACTGGTGAACGATTTCTATAATAACGGTCTG

CCGAGCAATCTGACCGCCAGCAGCAATCCGAGCCTGGATTATGGTTTTAAAGGTGCA

GAAATTGCAATGGCCAGCTATTGCAGCGAACTGCAGTATCTGGCAAATCCGGTTACCA

GCCATGTTCAGAGCGCAGAACAGCATAATCAGGATGTTAATAGCCTGGGTCTGATTAG

CAGCCGTAAAACCAGCGAAGCAGTTGATATTCTGAAACTGATGAGCACCACCTTTCT

GGTTGGTATTTGTCAGGCCGTTGATCTGCGTCATCTGGAAGAAAATCTGCGCCAGACC

GTGAAAAATACCGTTAGCCAGGTGGCAAAAAAAGTTCTGACCACCGGTATTAATGGT

GAACTGCATCCGAGCCGTTTTTGTGAAAAAGATCTGCTGAAAGTTGTGGATCGTGAA

CAGGTTTTTACCTATGTTGATGATCCGTGTAGCGCAACCTATCCGCTGATGCAGCGTCT

GCGTCAGGTTATTGTTGATCATGCACTGAGCAATGGTGAAACCGAGAAAAATGCAGT

GACCAGCATTTTTCAGAAAATCGGTGCATTTGAAGAGGAACTGAAAGCAGTTCTGCC

TAAAGAAGTTGAAGCAGCACGCGCAGCATATGGTAATGGTACGGCACCGATTCCGAA

TCGTATTAAAGAATGTCGTAGTTATCCGCTGTATCGTTTTGTTCGTGAAGAACTGGGCA

CCAAACTGCTGACAGGTGAAAAAGTTGTTAGTCCGGGTGAAGAATTCGATAAAGTTT

TTACCGCAATGTGCGAAGGCAAACTGATTGATCCTCTGATGGATTGTCTGAAAGAATG

GAATGGTGCCCCTATTCCGATTTGTTAA

Sequence of BdPAL1:

AAGGAGGAATAAACCATGGCAGGTAATGGTGCAATCAGCGAAAAAGATCCGCTGAAT

TGGGGTGCAGCAGCAGCCGAACTGACCGGTAGCCATCTGGATGAAGTTAAACGTATG

GTTGCACAGTTTCGTGAACCGGTTGTTAAAATTGAAGGTGCAAGCCTGCGTGTTGGT

CAGGTTGCAGCAGTTGCACAGGCAAAAGATGCAGCCGGTGTTAGCGTTGAACTGGA

CGAAGAAGCACGTCCTCGCGTTAAAGCAAGCAGCGAATGGATTCTGAGCTGTCTGGC

AGCAGGCGGTGATATTTATGGTGTTACCACCGGTTTTGGTGGCACCAGCCATCGTCGT

ACCAAAGATGGTCCGGCACTGCAGGTTGAACTGCTGCGTCATCTGAATGCAGGTATTT

TTGGCACCGGTAGTGATGGTCATAGCCTGCCTGCCGAAGTTACCCGTGCAGCAATGCT

GGTTCGCATTAATACCCTGCTGCAGGGCTATAGCGGTATTCGTTTTGAAATTCTGGAAG

CCATTACCAAACTGCTGAATACCGGTGTGAGCCCGTGTCTGCCGCTGCGTGGTACAAT

TACCGCAAGCGGTGATCTGGTTCCGCTGAGCTATATTGCAGGTCTGATTACCGGTCGT

CCGAATGCACAGGCCACCACCGCAGATGGTCGTAAAGTTGATGCAGCAGAAGCATTT

AAAGTTGCCGGTATTGAAGGCGGTTTTTTACCCTGAATCCGAAAGAAGGTCTGGCA

ATTGTTAATGGTACAAGCGTTGGTAGCGCACTGGCAGCAACCGTTCTGTTTGATTGTA

ATGTTCTGGCAGTTCTGAGCGAAGTGCTGAGCGCAGTTTTTGTGAAGTTATGAATGG

CAAACCGGAATTCACCGATCATCTGACCCATAAACTGAAACATCATCCGGGTAGCATT

GAAGCAGCAGCAATTATGGAACATATTCTGGCAGGTAGCAGCTTTATGAGCCATGCCA

AAAAAGTGAATGAAATTGATCCGCAGCTGAAACCGAAACAGGATCGTTATGCACTGC

GTACCAGTCCGCAGTGGCTGGGTCCGCAGATTGAAGTTATTCGTAGCGCAACCAAAA

GCATTGAACGTGAAGTTAATAGCGTGAATGATAACCCGGTTATTGATGTGCATCGTGG

TAAAGCACTGCATGGTGGTAATTTTCAGGGCACCCCGATTGGTGTTAGCATGGATAAT

ACCCGTCTGGCCATTGCAAATATTGGCAAACTGATGTTTGCCCAGTTTAGCGAACTGG

TGAATGAGTTTTATAACAATGGTCTGACCAGCAATCTGGCAGGCAGCCGTAATCCGAG

CCTGGATTATGGTTTTAAAGGCACCGAAATTGCCATGGCCAGCTATTGCAGCGAACTG

CAGTATCTGGCAAATCCGGTTACCAATCATGTTCAGAGCGCAGAACAGCATAATCAGG

ATGTTAATAGCCTGGGTTTAGTTAGCGCACGTAAAACCGCAGAAGCAGTTGATATTCT

GAAACTGATGAGCAGCACCTATATGGTTGCCCTGTGTCAGGCAGTTGATCTGCGTCAC

CTGGAAGAAAACATTAAAGCCAGCGTGAAAAATTGCGTTACCCAGGTTAGCAAAAA

AGTCCTGACCATGAATCCGACAGGTGATCTGAGCAGCGCACGTTTTAGCGAAAAATC

ACTGCTGACCGCAATTGATCGTGAAGCAGTTTTTAGCTATGCAGATGATGCATGTAGC

GCAAATTATCCGCTGATGCAGAAACTGCGTGCAGTTCTGGTTGATCATGCACTGACCA

GCTCAGGTGTTGATAATGCCGGTGAAAGCGAAGCCACCGTTTTTAGCAAAATCAACA

AATTCGAAGAGGAATTACGTGCAGCACTGCCTCGTGAAATTGAAGCCGCACGTGTTG

CATTTGAAAAAGGTACAGCACCGATTCCGAACCTGATTAAAGATAGCCGTAGCTTTCC

GCTGTATCGTTTTGTTCGTGAAGAACTGGGTTGTGTTTATCTGACCGGTGAGAAACTG

CTGTCACCGGGTGAAGAATGTAACAAAGTGTTTATTGGTATCAGCCAGGGTAAACTG

ATTGATCCGATGCTGGAATGTCTGAAAGAATGGAATGGTGAACCGCTGCCGATTAATG

TTGTTTAAGATCCGAGCTCGAGA

Sequence of BdPAL2:

TTGGAGGAATAAACCATGGAATGCGAAAATGGTCTGGTTGGTAGCCTGAATGGTGAA

GGTCTGTGTATGAGCGCACCGCCTCGTGCAGCAGATCCGCTGAATTGGGCAAAAACC

GCAGAAGAACTGGCAGGTAGCCATCTGGAAGAGGTGAAGTGAAAAATGGTTGCACA

GTTTCGTATGCCGCTGGTTAAAATTGAAGGTGCAACCCTGGGTATTGCACAGGTTGCA

GCAGTTGCAGCCGTGC@@

Sequence of BdPAL6:

AAGGAGGAATAAACCATGGCCTGTGAAAATGGTCAGGTTGCAGCAAATGGTATTTGT
ACCGCAATTCAGCATGCAGATCCGCTGAATTGGGGCAAAGCAGCAGAAGCACTGACC
GGTAGCCATCTGGAAGAGGTTAAACGTATGGTTGCAGAATATCGTCAGCCGGTTGTTA
CCATTGAAGGTGCAAGCCTGAGCATTGCAAAAGTTGCAGCAGTTGCCGCAGCCGGTG
AAGCACAGGTTCAGCTGGATGAAAGCGCACGTGAACGTGTTAAAGCAAGCAGCGAT
TGGGTTATGGATAGCATGGCAAATGGTGTTGATAGCTATGGTGTTACCACCGGTTTTGG
TGCAACCAGCCATCGTCGTACCAAAGAAGGTGGCGCACTGCAGCGTGAACTGATTCG
TTTTCTGAATGCCGGTGCATTTGGCACCGGTAGTGATGGTCATGTTCTGCCTGCGGGT
GCAACCCGTGCAGCAATGCTGGTTCGCATTAATACCCTGCTGCAGGGCTATAGCGGTA
TTCGTTTTGAAATTCTGGAAGCCATTGCCAAACTGCTGAATGCAAATGTTACCCCGTG
TCTGCCGCTGCGTGGCACCATTACCGCAAGCGGTGATCTGGTTCCGCTGAGCTATATT
GCAGGTCTGGTTACCGGTCGTGAAAATAGCGTTGCAGTTGCACCGGATGGTAGCAAA
GTTAATGCAGCCGAAGCCTTTAAAATCGCAGGTATTCATGGTGGCTTTTTTGAACTGC
AGCCTAAAGAAGGTCTGGCAATGGTTAATGGCACCGCAGTTGGTAGCGGTCTGGCGA
GCACCGTTCTGTTTGATGCCAATGTTCTGGCAGTTATGGCAGAAGTTATTAGCGCAGT
TTTTTGCGAAGTGATGAATGGCAAACCGGAATTTACCGATCATCTGACCCATAAACTG
AAACATCATCCGGGTCAGATTGAAGCAGCAGCAATTATGGAACATATCCTGGAAGGTA
GCAGCTATATGAAACTGGCAAAAAAACTGGGCGATCTGGATCCTCTGATGAAACCGA
AACAGGATCGTTATGCACTGCGTACCAGTCCGCAGTGGCTGGGTCCGCAGATCGAAG
TTATTCGTGCAGCCACCAAAAGCATTGAACGTGAAATTAACAGCGTGAATGACAACC
CGCTGATTGATGTTAGCCGTGGTAAAGCAATTCATGGCGGTAATTTTCAGGGCACCCC
GATTGGTGTTAGCATGGATAATACCCGTCTGGCACTGGCAGCAATTGGTAAACTGATG

TTTGCACAGTTTAGCGAACTGGTGAACGATTTCTATAATAACGGTCTGCCGAGTAATC

TGAGCGGTGGTCGTAATCCGAGCCTGGATTATGGTTTTAAAGGTGCAGAAATTGCAAT

GGCCAGCTATTGCAGCGAGCTGCAGTTTCTGGGTAATCCGGTTACCAATCATGTTCAG

AGCGCAGAACAGCATAATCAGGATGTTAATAGCCTGGGTCTGATTAGCGCACGTAAAA

CCGCAGAAGCAATTGAAATCCTGAAACTGATGACCAGCACCTTTCTGGTTGCCCTGT

GTCAGGCAATTGATCTGCGTCATATTGAAGAAAATGTGAAAAGCGCAGTTACCAGCT

GTGTTCGTGCAGTTGCCAAAAAAACCCTGAGCACCAATAGTGCCGGTGGTCTGCATG

TTGCCCGTTTTAGCGAAAAAGATCTGATCCAAGAAATTGATCGTGAAGCCGTTTTTGC

ATATGCCGATGATCCGTGTAATCCGAATTATCCGTTAATGAAAAAGCTGCGTGGTGTTC

TGGTTGAACGTGCACTGGCCAATGGTGTGGCAGAATTTGATGCAGAAACCAGCGTTT

TTGCCAAAGTTGCGCGTTTTGAAGAGGAACTGCGTGCAGCCCTGCCGGTTGCCGTTG

AAGCCGCACGTGCAGCCGTTGAAAGCGGTACAGCGGAAGCACCGAATCGTATTGCC

GAATGTCGTAGCTATCCGCTGTATCGTTTTGTTCGTCAAGAACTGGGCACCGTTTATCT

GACCGGTGAAAAAACCCGTAGTCCGGGTGAAGAACTGAATAAAGTTCTGGTGGCAA

TTAACCAGGGCAAACATATTGATCCGCTGCTGGAATGTCTGAAAGAATGGAATGGTG

AACCGCTGCCGATTTGTTAAGATCCGAGCTCGAGA

Sequence of BdPAL8:

AAGGAGGAATAAACCATGGAATATGAAAATGGTCACGCAGCAACCTATGGTGATGGT
CTGTGTGTTGCAGCACCGCTGGCACCGCGTGCAGATCCGCTGAATTGGGGTAAAGCA
GCAGAAGAACTGAGCGGTAGCCATCTGGATGCAGTTAAACGTATGGTTGAAGAATAT
CGTCGTCCGGTTGTTAAAATGGAAGGTGCAAGCCTGACCATTGCACAGGTTGCAGCA
GTTGCAGCCGGTGCCGAAGCACGTGTTGAACTGGATGAAAGCGCACGTGGTCGTGTT
AAAGAAAGCAGCGATTGGGTTATGAATAGCATGATGAATGGCACCGATAGTTATGGTG
TTACCACCGGTTTTGGTGCAACCAGCCATCGTCGTACCAAAGAAGGTGGCGCACTGC
AGCGTGAACTGATTCGTTTTCTGAATGCCGGTGCATTTGGCACCGGTGAAGATGGTCA
TGTTCTGCCTGCAGCAGCAACCCGTGCAGCAATGCTGGTTCGTGTTAATACCCTGCTG
CAAGGTTATAGCGGTATTCGTTTTGAAATCCTGGAAACCATTGCAACCCTGCTGAATG
CAAATGTTACCCCGTGTCTGCCGCTGCGTGGCACCATTACCGCAAGCGGTGATCTGGT
TCCGCTGAGCTATATTGCAGGTCTGGTTACCGGTCGTCCGAATAGCGTTGCAACCGCA
CCGGATGGTCGTAAAGTTAATGCAGCCGAAGCCTTTAAAATCGCAGGTATTCAGCATG
GCTTTTTTGAACTGCAGCCTAAAGAAGGTCTGGCAATGGTTAATGGTACAGCAGTTG
GTAGCGGTCTGGCCAGCATGGTTCTGTTTGAAGCAAATATTCTGGGTGTTCTGGCAGA
AGTTCTGAGCGCAGTTTTTTGTGAAGTTATGAATGGTAAACCGGAATTCACCGATCAT
CTGACCCATAAACTGAAACATCATCCGGGTCAGATTGAAGCAGCAGCCATTATGGAA
CATATTCTGGAAGGTAGCAGCTATATGATGCTGGCAAAAAAACTGGGCGAACTGGATC
CTCTGATGAAACCGAAACAGGATCGTTATGCACTGCGTACCAGTCCGCAGTGGCTGG
GTCCGCAGATCGAAGTTATTCGTGCAGCCACCAAAAGCATTGAACGTGAAATTAACA
GCGTGAATGACAACCCGCTGATTGATGTTAGCCGTGGTAAAGCAATTCATGGTGGTAA
TTTTCAGGGCACCCCGATTGGTGTTAGCATGGATAATACCCGTCTGGCCATTGCAGCA

ATTGGTAAACTGATGTTTGCACAGTTTAGCGAACTGGTGAACGATTTCTATAATAACG

GTCTGCCGAGCAATCTGAGCGGTGGTCGTAATCCGAGCCTGGATTATGGTTTTAAAGG

TGCAGAAATTGCAATGGCGAGCTATTGCAGCGAGCTGCAGTTTCTGGGTAATCCGGTT

ACCAATCATGTTCAGAGCGCAGAACAGCATAATCAGGATGTTAATAGCCTGGGTCTGA

TTAGCAGCCGTAAAACCGCAGAAGCAATTGATATTCTGAAACTGATGAGCAGCACCT

TTCTGGTTGCCCTGTGTCAGGCAATTGATCTGCGTCATCTGGAAGAAAATGTTCGTAG

CGCAGTGAAAAATTGTGTGACCACCGTTGCACGTAAAACCCTGAGCACCAATGTGAA

TGGTCATCTGCATAATGCACGCTTTTGTGAAAAAGATCTGCTGCTGACAATTGATCGT

GAAGCAGTTTTTGCATATGCCGATGATCCGTGTAGCGCAAATTATCCGCTGATGCAGA

AAATGCGTGCAGTTCTGGTTGAACATGCACTGGCAAATGGTGAAGCAGAACGTGATG

TTGAAACCAGCGTTTTTGCCAAACTGGCAGCATTTGAACAAGAACTGCGTGCCGTGC

TGCCGAAAGAAGTTGAAGCCGCACGCGCAGCCGTTGAAAATGGCACAGCGACCAAA

CAGAATCGTATTGCAGAATGTCGTAGCTATCCGCTGTATCGTTTTGTTCGTGAAGAACT

GGGCACCGAATATCTGACCGGTGAAAAAACCCGTAGTCCGGGTGAAGAAGTGGATAA

AGTTTTTGTTGCAATGAACCAGGGCAAACATATTGATGCACTGCTGGAATGTCTGAAA

GAATGGAATGGTGAACCGCTGCCGCTGTGTTAAGATCCGAGCTCGAGA

Sequence of FDC1:

CATATGGGAATTCGAAAAGAGGTATATATTAATGCGTAAACTGAATCCGGCACTGGAA

TTTCGTGATTTTATTCAGGTTCTGAAAGATGAGGATGACCTGATTGAAATCACCGAAG

AAATTGATCCGAATCTGGAAGTTGGTGCCATTATGCGTAAAGCATATGAAAGCCATCT

GCCTGCACCGCTGTTTAAAAACCTGAAAGGTGCAAGCAAAGACCTGTTTAGCATTCT

GGGTTGTCCGGCAGGTCTGCGTAGCAAAGAAAAAGGTGATCATGGTCGTATTGCCCA

TCATCTGGGTTTAGATCCGAAAACCACCATCAAAGAGATCATCGATTATCTGCTGGAA

TGCAAAGAGAAAGAACCGCTGCCTCCGATTACCGTTCCGGTTAGCAGCGCACCGTGT

AAAACCCATATTCTGAGCGAAGAAAAAATCCATCTGCAGAGCCTGCCGACACCGTAT

CTGCATGTTTCAGATGGTGGTAAATATCTGCAGACCTATGGTATGTGGATTCTGCAGAC

ACCGGACAAAAAATGGACCAATTGGAGCATTGCACGTGGTATGGTTGTTGATGATAA

ACATATTACCGGTCTGGTGATTAAACCGCAGCATATTCGTCAGATTGCAGATAGCTGGG

CAGCAATTGGTAAAGCCAATGAAATTCCGTTTGCACTGTGTTTTGGTGTTCCGCCTGC

AGCAATTCTGGTGAGCAGCATGCCGATTCCGGAAGGTGTTAGCGAAAGCGATTATGTT

GGTGCGATTCTGGGTGAAAGCGTTCCGGTGGTTAAATGTGAAACCAATGATCTGATG

GTTCCGGCAACCAGCGAAATGGTTTTTGAAGGCACCCTGAGCCTGACCGATACACAT

CTGGAAGGTCCGTTTGGTGAAATGCATGGTTATGTGTTTAAAAGCCAGGGTCATCCGT

GTCCGCTGTATACCGTTAAAGCAATGAGCTATCGTGATAATGCAATTCTGCCGGTGAG

CAATCCGGGTCTGTGTACCGATGAAACCCATACACTGATTGGTAGCCTGGTTGCCACC

GAAGCAAAAGAACTGGCAATTGAAAGCGGTCTGCCGATTCTGGATGCATTTATGCCG

TATGAAGCACAGGCACTGTGGCTGATTCTGAAAGTTGATCTGAAAGGACTGCAGGCA

CTGAAAACCACACCGGAAGAATTTTGTAAAAAAGTGGGCGATATCTACTTCCGTACC

AAAGTTGGTTTTATCGTGCATGAAATTATTCTGGTGGCCGATGATATCGACATCTTCAA

CTTTAAAGAAGTGATTTGGGCATACGTGACCCGTCATACACCGGTTGCAGATCAGATG

GCATTTGATGATGTTACCAGCTTTCCGCTGGCACCGTTTGTTAGCCAGAGCAGCCGTA

GCAAAACAATGAAAGGTGGCAAATGTGTGACCAACTGTATTTTTCGTCAGCAGTATG

AACGCAGCTTCGATTATATCACCTGTAACTTTGAAAAGGGCTATCCGAAAGGTCTGGT

GGATAAAGTTAATGAAAACTGGAAACGCTACGGCTATAAATAATGGCTGTTTTGGCGG
